# 2-methyl butyramide, a previously identified urine biomarker for *Ascaris lumbricoides*, is not present in infected Indonesian individuals

**DOI:** 10.1186/s13071-017-2600-z

**Published:** 2017-12-29

**Authors:** Ole Lagatie, Emmanuel Njumbe Ediage, Jeroen A. Pikkemaat, Yenny Djuardi, Lieven J. Stuyver

**Affiliations:** 1 Janssen Diagnostics, Janssen R&D, Turnhoutseweg 30, 2340 Beerse, Belgium; 2 Bioanalysis, Janssen R&D, Turnhoutseweg 30, 2340 Beerse, Belgium; 3 Pharmaceutical Development and Manufacturing Sciences, Janssen R&D, Turnhoutseweg 30, 2340 Beerse, Belgium; 4 Department of Parasitology, Faculty of Medicine, Universitas Indonesia, Jakarta, Indonesia

**Keywords:** *Ascaris lumbricoides*, Soil-transmitted helminths, Biomarker, Urine, 2-methyl butyramide, 2-methyl valeramide

## Abstract

**ᅟ:**

Previous reports suggest that the 2-methyl butyramide and 2-methyl valeramide metabolites of *Ascaris lumbricoides* in urine of infected individuals could be considered as urinary biomarkers for active infection. We have developed an LC-MS method with a detection limit of 10 ng/mL using synthetic chemicals as reference material. Urine samples (*n* = 21) of infected individuals were analyzed for the presence of these metabolites, but they were not detected in any of the samples. Furthermore, the recorded ^1^H-NMR spectrum for reference 2-methyl butyramide did not match with the spectrum that was described for the *Ascaris* metabolite. Based on these two observations, we concluded that the urinary biomarkers that were detected for *A. lumbricoides* infection are not 2-methyl butyramide nor 2-methylvaleramide. New discovery efforts will be required to identify the structure of these metabolite biomarkers in urine of infected individuals.

**Trial registration:**

Urine samples used in this study were collected as part of a clinical trial with trial number ISRCTN75636394 (12 November 2013).

**Electronic supplementary material:**

The online version of this article (10.1186/s13071-017-2600-z) contains supplementary material, which is available to authorized users.

## Letter to the Editor

Infection with soil-transmitted helminth (STH) parasites, such as *Ascaris lumbricoides*, hookworm and *Trichuris trichiura*, is a major health issue in large parts of the world, causing severe morbidity, especially in children. In addition to the development of new treatment options, there is also a growing need for improved diagnostics. Currently, screening (and diagnostic) procedures that are being used in the field are either based on microscopic examination of stool samples or molecular detection in stool [1–3]. Both procedures are time-consuming, require trained personnel and specific laboratory infrastructure. However, the STH community has begun exploring opportunities to strengthen the monitoring of drug donation program impact, and success of these monitoring efforts will most likely depend on the introduction of new diagnostic tools for non-stool based biomarkers. In a search for such non-stool based biomarkers for STH infection, the article of Hall & Romanova is of particular interest [4]. In this paper, the authors described the identification of 2-methyl butyramide (2-MBA) and 2-methyl valeramide (2-MVA) in urine as promising biomarkers for infection with *A. lumbricoides*. Furthermore, there seems to be a correlation with the metabolite concentration and worm burden. An extended literature search did not generate any follow-up research, neither by the authors themselves, nor by other groups who have tried to confirm these findings. Therefore, we developed a liquid chromatography tandem mass spectrometry (LC-MS) method for the quantification of 2-MBA and 2-MVA levels with a limit of quantification of 10 ng/ml urine, and using synthetic 2-MBA (Sigma-Aldrich, Overijse, Belgium) and 2-MVA (AKos GmbH, Steinen, Germany) as reference material. Identity and purity of the reference materials was certified by the supplier. The method showed linearity over the range from 10 ng/ml up to 20 μg/ml with a precision ranging from 0.7 to 4.4%. Stability of 2-MBA was investigated and it was found to be stable (< 15% difference) for 24 h at 4 °C, room temperature and 50 °C. Furthermore, this molecule was observed to be light stable (4 h at 250 W/m^2^).

Since the authors described that the concentration of 2-MBA can be estimated to be about 0.006 g/100 ml urine/100 g wet weight of worms, we assumed that an infection with a single adult worm (weighing *c.*3 g) should result in urinary levels of 2 μg/ml, which is 200-fold higher than the limit of quantification of our LC-MS method. A biorepository of urine samples (*n* = 21, stored frozen) collected from individuals living in the Nangapanda area, Flores Island, Indonesia, with stool-based qPCR-confirmed infection with *A. lumbricoides*, or multiple infection with *A. lumbricoides* and *T. trichiura* or hookworm were analyzed for the presence of 2-MBA and 2-MVA [5]. In contrast to the published results, no detectable levels of 2-MBA or 2-MVA were found in any of the samples.

In the 1990 published report [4], the identification of the two urinary metabolites was mainly based on Proton Nuclear Magnetic Resonance (^1^H-NMR) Spectroscopy. To confirm the ^1^H-NMR description, we recorded a ^1^H-NMR spectrum of the 2-MBA reference material (Additional file 1). For 2-MVA no spectrum was recorded as insufficient amounts of this reference material were available. The reference material spectrum of 2-MBA was compared with the originally described spectrum but the data demonstrated that the spectrum described for the metabolite isolated from urine did not overlay with the reference spectrum of 2-MBA. Furthermore, the ^1^H-NMR spectrum we recorded matches with the theoretically expected peaks for this molecule, while this is not the case for the spectrum originally described.

In conclusion, we have shown that the urinary metabolite that was found to be a promising biomarker for infection with *A. lumbricoides* is not 2-methyl butyramide. The original ^1^H-NMR and infrared spectrum was not disclosed by the authors and only a brief description of the observed shifts and absorption bands is available which is insufficient to confidently deduce the chemical structure of this biomarker. We have contacted the original authors to get more information on the spectra but unfortunately none of them were able to give more information than what was available in the original publication. Hence, the question remains unanswered on the true nature of these biomarkers. Additional discovery research will be needed to isolate and characterize these unidentified metabolites from urine of STH infected individuals. If confirmed and successfully identified, these metabolites are promising biomarkers that can be used for the development of a non-stool based diagnostic monitoring tool for STH, similar to the Point-of-Care Circulating Cathodic Antigen (POC-CCA) test for *Schistosoma mansoni* [6, 7].

## Additional file

Additional file 1: 1H-NMR Spectrum of 2-methyl butyramide. (PPTX 230 kb)

## Abbreviations

^1^H-NMR: Proton nuclear magnetic resonance
2-MBA: 2-methyl butyramide
2-MVA: 2-methyl valeramide
LC-MS: Liquid chromatography coupled to mass spectrometry
STH: Soil-transmitted helminths
