# Supplementary figures and images for: 2-methyl butyramide, a previously identified urine biomarker for Ascaris lumbricoides, is not present in infected Indonesian individuals
Source: Parasit Vectors. 2017 Dec 29;10:629. doi: 10.1186/s13071-017-2600-z (PMC5747143; doi:10.1186/s13071-017-2600-z)

## Slide 1
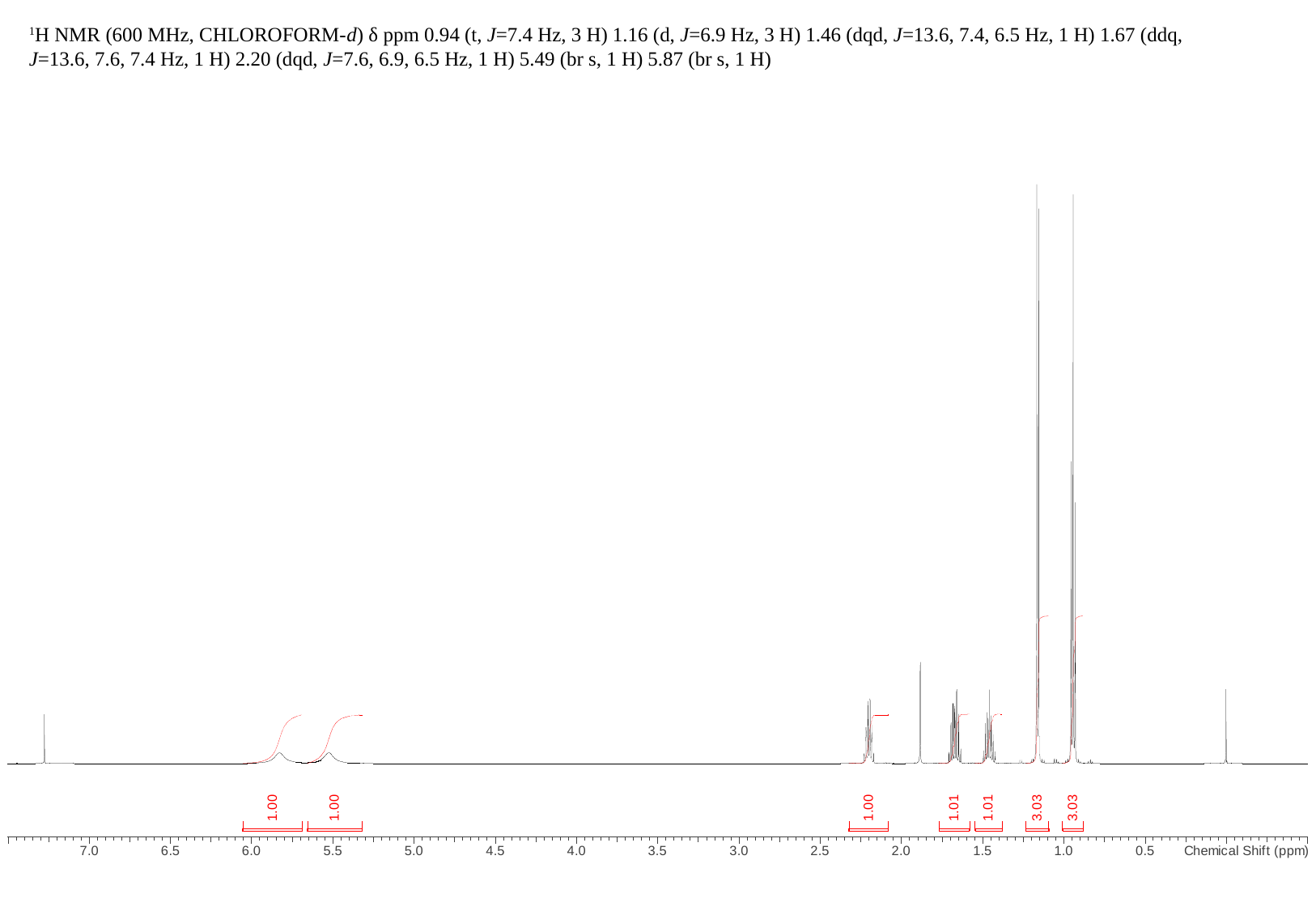

1H NMR (600 MHz, CHLOROFORM-d) δ ppm 0.94 (t, J=7.4 Hz, 3 H) 1.16 (d, J=6.9 Hz, 3 H) 1.46 (dqd, J=13.6, 7.4, 6.5 Hz, 1 H) 1.67 (ddq, J=13.6, 7.6, 7.4 Hz, 1 H) 2.20 (dqd, J=7.6, 6.9, 6.5 Hz, 1 H) 5.49 (br s, 1 H) 5.87 (br s, 1 H)

Supplement: Additional file 1: — 1H-NMR Spectrum of 2-methyl butyramide. (PPTX 230 kb) [file 13071_2017_2600_MOESM1_ESM.pptx]
